# Editorial: Self-Organization in the Nervous System

**DOI:** 10.3389/fnsys.2017.00069

**Published:** 2017-09-26

**Authors:** Yan M. Yufik, Biswa Sengupta, Karl Friston

**Affiliations:** ^1^Virtual Structures Research Inc., Potomac, MD, United States; ^2^Department of Bioengineering, Imperial College London, London, United Kingdom; ^3^Institute of Neurology, Wellcome Trust Centre for Neuroimaging, London, United Kingdom

**Keywords:** self-organization, neural circuits, variational inference, bayesian inference, dynamical systems theory

“Self-organization is the spontaneous—often seemingly purposeful—formation of spatial, temporal, spatiotemporal structures, or functions in systems composed of few or many components. In physics, chemistry and biology self-organization occurs in open systems driven away from thermal equilibrium” (Haken, Scholarpedia). The contributions in this special issue aim to elucidate the role of self-organization in shaping the cognitive processes in the course of development and throughout evolution, or “from paramecia to Einstein” (Torday and Miller). The central question is: what self-organizing mechanisms in the human nervous system are common to all forms of life, and what mechanisms (if any) are unique to the human species?

Over the last several decades, the problem of self-organization has been at the forefront of research in biological and machine intelligence (Kohonen, 1989; Kauffman, 1993; Pribram, 1994, 1996, 1998; Kelso, 1997; Camazine et al., 2003; Zanette et al., 2004; Haken, 2010, 2012, and others). The articles collected in this issue present recent findings (and ideas) from diverse perspectives and address different facets of the problem. Two features of this collection might be of particular interest to the reader: (i) the scope of discussion is broad, stretching from general thermodynamic and information-theoretic principles to the expression of these principles in human cognition, consciousness and understanding and (ii) many of the ideas speak to a unifying perspective outlined below. In what follows, we will preview the collection of papers in this special issue and frame them in terms of a unified approach to self organization—leaving the reader to judge the degree to which subsequent articles are consistent with or contradict this framework.

Living organisms must regulate flows of energy and matter through their boundary surfaces to underwrite their survival. Cognitive development is the product of progressive fine-tuning (optimization) of regulatory mechanisms, under the dual criteria of minimizing surprise (Friston, 2010; Sengupta et al., 2013, 2016; Sengupta and Friston, 2017) and maximizing thermodynamic efficiency (Yufik, 2002, 2013). The former implies reducing the likelihood of encountering conditions impervious to regulation (e.g., inability to block inflows of destructive substances); the latter implies maintaining net energy intakes above some survival thresholds. Energy is expended in regulatory processes formed in the course of self-organization and predicated on lowering thermodynamic entropy “on the inside” and transporting excessive entropy (heat) “to the outside.” Efficient regulation requires mechanisms that necessarily incorporate models of the system and its relation to environment (Conant and Ashby, 1970). Primitive animals possess small repertoires of genetically fixed, rigid models, while—in more advanced animals—the repertoires are larger and their models become more flexible; i.e., amenable to experience-driven modifications. Both the evolutionary and experience-driven modifications are forms of statistical learning: models are sculpted by external feedback conveying statistical properties of the environment. Human learning mechanisms, although built on the foundation of statistical learning, depart radically from conventional (e.g., machine) learning: the implicit models become amenable to self-directed composition and modification based on interoceptive, as opposed (or in addition) to exteroceptive, feedback (Yufik, 1998). Interoceptive feedback underlies the feeling of grasp, or understanding that accompanies the organization of disparate “representations” into cohesive structures amenable to further operations (mental modeling). The work of mental modeling requires energy; consciousness is co-extensive with deliberate (attentive, focused) application of energy (“cognitive effort”) in carrying out that work. Learning with understanding departs from statistical (machine) learning in three ways: (i) mental models anticipate experiences, as opposed to be shaped by them (e.g., the theory of relativity originated in gedanken experiments); (ii) feedback conveys properties of implicit models (coherence, simplicity, validation opportunities the models afford, etc.) and (iii) manipulating (executing or inverting) models enables efficient exchange with the environment, under conditions with no precedents (and thus no learnable statistical representation) (Yufik, 2013). Regulation of this sort—based on statistical learning—faces a challenging complexity. As the number of regulated variables grows; energy demands can quickly become unsustainable. Using self-organization to implement the process of “understanding” (i.e., composing more general models) has the triple benefit of minimizing surprise, while averting complexity and advancing thermodynamic efficiency of regulatory processes into the vicinity of theoretical limits.

Annila argues that the most fundamental function performed by the nervous system is shared by all open systems and entails a generation of entropy, by extracting high-grade free energy from the environment and returning low-grade energy. As dictated by the second law of thermodynamics, cognitive processes seek out opportunities (paths) for consuming free energy in the least time. Evolution obtains progressively more efficient mechanisms for detecting and exploiting free energy deposits, culminating in consciousness that emerges in systems pertaining to the ability to “integrate various neural networks for coherent consumption of free energy…” (Annila, this issue).

Street reviews discussions in the literature that examine the tension between—and synthesis of—information-theoretic and thermodynamics-motivated conceptualizations of brain processes. Tensions are rooted in the theory of information, designed to allow analysis of information transfer, irrespective of the physical processes that mediate transfer. Synthesis is necessitated by considerations of energy costs incurred in neuronal signaling. A consensus is anticipated, within a theoretical framework that views cognitive development as self-organization in the nervous system—seeking to minimize surprise, while incurring minimum energy costs.

Torday and Miller discuss the conceptual framework needed for tracing evolution of the mammalian brain “from paramecia to Einstein.” The framework encompasses three key notions: (i) complex multicellular organisms share fundamental organizational properties, with precursors in unicellular forms of life, (ii) the most basic property is the ability to extract energy from the environment and dissipate heat in a manner enabling homeostasis and processing of information and (iii) evolutionary improvements in homeostasis, self-maintenance and information processing derive from increased cellular collaboration (coherence). Within this framework, “life is cognition at every scope and scale” and “any cognitive action as a form of cellular coherence can be better understood as both an information exchange and reciprocally then, as energy conversion and transfer” (Torday and Miller).

Campbell argues that Darwinian evolution can be expressed as a process of Bayesian updating. Conventionally, the ability to draw inferences and update Bayesian models has been attributed exclusively to (human) reasoning. The range of attribution can be expanded to include all organisms, by assuming that genotypes carry latent models of the environment receiving varying expressions in the phenotype. On that view, genetically transmitted models are the source of hypotheses (phenotype variations) subjected to confirmation (survival) or rejection (extinction) by the environment. Changes in the phenotype over somatic time and the genotype over evolutionary time minimize surprise thus increasing the likelihood of survival of individuals and the species.

Kozma and Freeman analyze alternations between highly organized (low entropy) and disorganized (high entropy) neuronal activities induced by visual stimuli. In rabbits implanted with ECoG arrays of electrodes fixed over the visual cortex, presentations of stimuli were accompanied by metastable patterns of synchronized activity—collapsing quickly into the background activity upon cessation of the stimuli. The authors define alternations between metastable patterns and disorganized firings as phase transitions and propose a “cinematic” theory of perception; treating alternations that spread across the cortex as successions of “frames” combined into perceptual units (percepts). Synchronized neuronal populations are identified with Hebbian assemblies, acting in a self-catalytic fashion: Interactions between assemblies maintain the cortex in the critical state, conducive to the emergence of organized (low entropy) structures, such as Hebbian assemblies.

Stankovski et al. present novel findings concerning the coherence of neuronal assemblies. Assemblies oscillate within characteristic frequency intervals, with cross-frequency coupling serving to integrate assemblies into functional networks that span distant regions in the brain. In this study, cross-frequency coupling functions were reconstructed from EEG recordings from human subjects in the state of rest, with the eyes either open or closed. They review early evidence that closing the eyes triggers an increase in coupling strength. A novel method of analysis then allows them to determine variations in coupling strength across frequency ranges: crucially, they find that increases in the strength of inter-assembly coupling are accompanied by narrowing variation envelopes.

Tang et al. recorded experience-induced changes in the connectivity of large-scale brain networks. Subjects were resting in a state of “mindfulness,” under minimal exposure to external stimuli. A comprehensive array of mathematical analyses was applied to the fMRI data. The analyses reveal statistically significant increases in connectivity between different brain areas. Many earlier studies have demonstrated increased connectivity in brain networks under external stimuli; however, according to this study, similar increases can be produced in the course of internally-induced, restful states.

Werbos and Davis review progress to date in modeling cognitive functions, focusing on the neural net model of learning employing back-propagation algorithms. Neural nets represent learning as the acquisition of desired mappings between input vectors (environmental conditions) and output vectors (desired responses), via iterative reduction of mapping errors. The model posits successions of calculations propagating forward and backward in the neuronal system, orchestrated by some global clock. Empirical substantiations of this model have been scarce—but new experimental findings and analysis are presented that speak to its biological plausibility.

Perlovsky's “physics in the mind” research program tries to define the principles of cognition in a rigorous way (a la Newtonian mechanics). Some principles are suggested including *mental modeling, vague representations, knowledge instinct, dynamic logic* and *dual hierarchy*. A *mental model* is the basic functional unit of cognition, *models* are *vague* (lacking detail), while sensory inputs are *crisp* (rich in detail). Acquiring knowledge involves reconciling models and inputs in a process driven by *knowledge instinct* and employing mechanisms of *dynamic logic*. Model hierarchy has a counterpart in linguistic hierarchy (hence, the *dual hierarchy*).

Newton analyzes composition of understanding and identifies three constituents: (i) imagery, (ii) the state of mental tension (surprise) caused by a novel situation and (iii) the state of tension resolution, provided by having worked out responses afforded by the situation. The feeling of having reached understanding (Aha!) precedes response execution and thus depends on factors other than external feedback (although failures can restore tension). Execution involves some forms of bodily activities; so “understanding” is anchored in the mechanisms that control such activities. Understanding can then expand via mapping new situations onto those that are already understood.

Yufik and Friston suggest that the same self-organization principle manifests in both the emergence of life and evolution of regulatory mechanisms sustaining life: Regions (subnets) in networks of interacting units (molecules, neurons) fold into bounded structures stabilized by boundary processes. Evolution expanded regulation mechanisms from conditioning to anticipatory planning—that is accomplished via self-directed composition and execution of *mental models*. Hebbian assemblies stabilized by boundary energy barriers (*neuronal packets*) are produced by folding and phase transition in neuronal networks and represent (model) persistent constellations of stimuli (*objects*). Variations in packet responses (changes in the composition of responding groups and the order of their firing inside the packet) represent *behavior*. “Understanding” accompanies the composition of *models* representing behavior coordination (*inter-object relations)*, as bi-directional (reversible) mapping between packets. Such reversible mapping underlies behavior prediction and explanation (retrodiction). Coordination establishes thermodynamic equilibrium in the volume of a model thus minimizing dissipation (costs) and enabling reversible execution. Expanding models and exploring new inputs necessary moves the system away from equilibrium. Regulation via anticipation and explanation is a uniquely human form of surprise minimization. The regulatory process is supported by verbalization and imagery but is driven by *modeling*. Arguably, mental modeling, i.e., coordination of packets (mental objects) in the mental space builds on the neuronal machinery engaged in coordinating limbs and objects in the physical space.

This concludes our brief survey of the articles offered in the special issue. To an outside observer, cars might appear to have the purpose of seeking out gas stations and converting fuel into heat and exhaust. A closer inspection will reveal intelligent regulators inside the cars (i.e., you and me) concerned with having enough fuel to reach the next station—and averting the “surprise” of finding the fuel tank empty. Other concerns—that contextualize this regulation—are the cost of fuel and the desire to keep the car running for the greatest distance possible. In the process, cars must maneuver in coordination with other cars, traffic rules and terrain. Such self-motivated, self-evidencing and self-regulated cars might be a plausible metaphor for minds embedded in a self-organizing nervous system.

## Author contributions

YY, BS, and KF contributed equally to this editorial.

### Conflict of interest statement

The authors declare that the research was conducted in the absence of any commercial or financial relationships that could be construed as a potential conflict of interest.
